# Role of multiheme cytochromes involved in extracellular anaerobic respiration in bacteria

**DOI:** 10.1002/pro.3787

**Published:** 2019-11-28

**Authors:** Marcus J. Edwards, David J. Richardson, Catarina M. Paquete, Thomas A. Clarke

**Affiliations:** ^1^ Centre for Molecular and Structural Biochemistry School of Biological Sciences and School of Chemistry, University of East Anglia Norwich UK; ^2^ Instituto de Tecnologia Química e Biológica António Xavier, Universidade NOVA de Lisboa Oeiras Portugal

## Abstract

Heme containing proteins are involved in a broad range of cellular functions, from oxygen sensing and transport to catalyzing oxidoreductive reactions. The two major types of cytochrome (*b*‐type and *c*‐type) only differ in their mechanism of heme attachment, but this has major implications for their cellular roles in both localization and mechanism. The *b*‐type cytochromes are commonly cytoplasmic, or are within the cytoplasmic membrane, while *c*‐type cytochromes are always found outside of the cytoplasm. The mechanism of heme attachment allows for complex *c*‐type multiheme complexes, having the capacity to hold multiple electrons, to be assembled. These are increasingly being identified as secreted into the extracellular environment. For organisms that respire using extracellular substrates, these large multiheme cytochromes allow for electron transfer networks from the cytoplasmic membrane to the cell exterior for the reduction of extracellular electron acceptors. In this review the structures and functions of these networks and the mechanisms by which electrons are transferred to extracellular substrates is described.

## 1 BACKGROUND

All organisms conserve energy through electron transfer reactions. Phototropic organisms utilize light energy to move an electron to a low potential redox center from a high potential redox center. The subsequent movement of the electron through a chain of increasingly positive potential redox centers then occurs spontaneously. The energy released at each stage is used to generate a proton motive force, which then drives the formation of adenosine triphosphate (ATP). Chemotrophic organisms also couple the energy released by the oxidation of reduced substrates to generate a proton‐motive force. The electrons released again make their way through a succession of redox proteins that ultimately conserve energy through ATP, either through substrate level phosphorylation or through the generation of a proton motive force that ultimately powers ATP synthase. These catabolic processes all require the transfer of electrons from one redox center to another, either through inter‐ or intra‐protein electron transfer, or by both processes.

Typically, metals are required for biological redox reactions. A range of redox‐active metals can function as the catalytic sites on redox enzymes, most commonly iron, molybdenum, copper and tungsten, but also including cobalt and manganese. As iron is the most abundant transition metal in the earth's crust it is unsurprising that proteins containing multiple redox centers are abundant in iron. The redox centers that allow electrons to move between these centers, or store electrons for reactions requiring multiple electrons are typically composed of iron, either in the form of iron–sulfur clusters or heme groups. Iron–sulfur clusters are a more ancient form of redox center and are typically oxygen sensitive, so organisms must either retreat to anaerobic environments, or bury the cofactors deep inside proteins that are expressed under aerobic conditions, such as the mitochondrial NADH dehydrogenases.^1^, ^2^ Iron–sulfur clusters are a remarkably diverse range of cofactors whose structure, function, and evolution have been reviewed in great detail elsewhere.^3^, ^4^

Heme cofactors are a more recent evolution of redox center that are more oxygen tolerant than iron–sulfur clusters. There are several different types of heme cofactor, including heme *a*‐, *d*_*1*_‐, *o*‐, and siroheme but the most common forms are bound as part of *b*‐ and *c*‐type cytochromes.^5^ For these cytochromes the cofactor contains a single iron atom contained within a protoporphyrin IX molecule, which consists of a porphyrin ring with two propionate groups and two vinyl groups. The heme iron is coordinated by four imidazole ligands and one or two axial ligands formed by amino acid sidechains, more commonly histidine or methionine, or more rarely cysteine, lysine, or tyrosine. In some cases, particularly if the heme is a catalytic site of the enzyme, the sixth ligand is not contributed by the polypeptide but by an exogenous ligand such as water, hydroxide or an enzymatic substrate.

The key difference between *b*‐ and *c*‐type cytochromes lies in the way in which the protoporphrin IX molecule is associated with the polypeptide chain. In the former it is a noncovalent interaction, while in the latter it is a covalent interaction. The most common form of heme found in biological proteins is the *b‐*type heme, which is typically found in globin‐like and cytoplasmic heme proteins. In a *b*‐type cytochrome the heme inserts into a hydrophobic crevice of the protein structure with the propionates facing the protein surface and the axial ligands helping to position the heme within the hydrophobic cavity. *b*‐type cytochromes include electron transporters such as cytochrome b_5_, catalytic enzymes such as such as cytochrome P450 or globins that function as oxygen carriers or sensors.

*c*‐type cytochromes commonly function as electron transfer proteins or as oxidoreductive enzymes that catalyze the reduction of reactive substrates such as nitrite, sulfite, peroxide, or hydroxylamine. After translation of a c‐type cytochrome polypeptide, the unfolded apocytochrome is transported across the cytoplasmic membrane by the Sec system, after which CXXCH motifs within the amino acid sequence are recognized by one of four different cytochrome maturation systems that covalently attach a heme group to the two cysteine residues via thio‐ether linkages between the cysteine sulfur and the vinyl groups of the heme.^6^ This covalent attachment forces the histidine adjacent to the cysteine to form the distal ligand to the iron atom. Each peptide can carry several of CXXCH motifs, allowing for multiple hemes to be attached to each protein.^7^ CXXCH is the most common cytochrome maturation motif, although the maturation system is flexible in attaching hemes with motifs containing more than two amino acids between the cysteines.^8^ For example the octaheme MccA of *Wollinella succinogenes* includes a CX_15_CH heme binding site.^9^ Recently a “contracted” heme binding motif of CKCH was identified in a tetraheme protein that is part of the hydrazine synthase apparatus of annamox bacteria *Kuenenia stuttgartiensis*.^10^ However, it is clear that the adjacent cysteine‐histidine motif is critical, such that a similar motif CXXCK requires a separate *ccm* maturation system to covalently attach the heme to the active site of cytochrome *c* nitrite reductase NrfA. This allows the coordination of a lysine to the distal side of the active site heme.^11^ In addition, the CX_15_CH motif of *Wolinella succinogenes* MccA also required a dedicated heme attachment apparatus.^9^

Genomic and metagenomic sequencing has revealed many genes that would encode for cytochromes containing multiple covalently attached hemes. The largest identified so far is a hypothetical protein from *Agromonas oligotrophica* S58 that contains 86 CXXCH motifs. Other examples include a protein from *Desulfuromonas soudanensis* and *Thermincola ferriacetica* predicted to contain 69 hemes and 58 hemes respectively.^12^, ^13^, ^14^ The roles of these cytochromes have not been determined so it is unclear why it is necessary to generate a single protein that would contain so many hemes, or even whether these proteins can be expressed with a full complement of hemes at all.

The range of redox potentials achievable by both *b*‐type and *c*‐type cytochromes are similar,^15^ suggesting the reason for evolution of both *b‐* and *c‐*type hemes is unlikely to be catalytic, but could be to do with the increased stability and higher capacity for “heme loading” in *c‐*type cytochromes. To better understand the differences between *b*‐ and *c*‐type cytochromes we performed a bioinformatic analysis of proteins containing either noncovalently or covalently attached hemes. While it is possible to identify possible *c*‐type cytochromes based on the CXXCH motif and signal peptides for membrane translocation, there is no clear motif that unambiguously assigns a protein as a *b*‐type cytochrome. To compare the different classes of *b*‐ and *c*‐ type heme containing cytochrome we therefore searched the RCSB Protein Data Bank for structures of proteins known to contain either *b‐*type (residue code HEM) or *c‐*type (residue code HEC) heme ligands. Previous studies have reported such ratios in terms of amino acid number per heme,^16^, ^17^ however this does not account for the size of amino acids in the protein. Using the monomer‐weight per heme group gives a value for the amount of protein, including sidechains, that is required to support each heme group.

**Figure 1:**
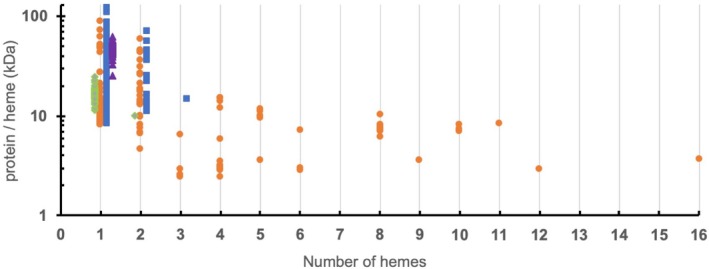
Comparison of normalized sizes of proteins containing one or more hemes. The relative molecular weight of protein (in kiloDaltons, kDa) *per* heme group is shown against number of hemes. Protein structures with less than 50% homology predicted to be either *b*‐ or *c*‐type cytochromes were obtained from the databank and sorted according to number of hemes contained within a protein monomer. Green diamonds represent globins, purple triangles represent proteins with thiolate ligated hemes (typically P450‐like). Blue squares represent other *b*‐type cytochromes, while orange circles represent *c*‐type cytochromes

After obtaining a list of 510 structures with less than 50% amino acid sequence similarity, we checked each one to determine whether it was a *b*‐type or *c*‐type by checking hemes for covalent cysteine ligands. *b*‐type cytochromes comprise approximately 70% of the total number of structures, while *c*‐type cytochromes comprise the remaining 30% of heme containing proteins. Of these heme‐containing structures approximately 68% are predicted to contain a single heme, 15% to contain two hemes and the remaining 16% contain 3–16 hemes per monomer. We split the *b‐*type hemes into three classes, general *b*‐type, globin, and cysteine ligated (typically P450 monoxygenases) and then determined the relative molecular weight per heme group of each protein structure in the protein data bank (Figure 1).

### 1.1 *Monoheme cytochromes*

**Figure 2:**
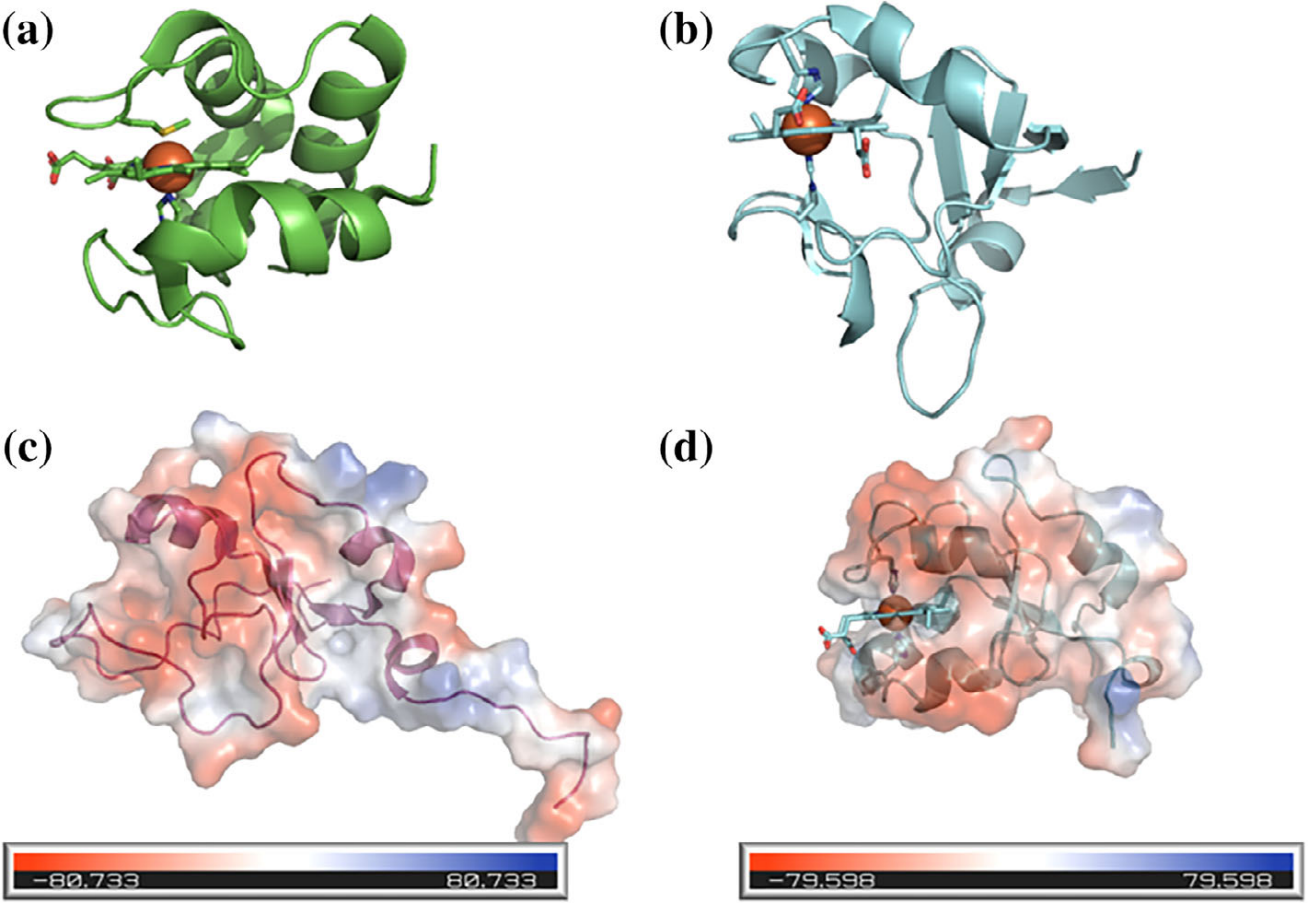
Cartoon views of cytochrome structures. (a) 7.1 kDa structure of cytochrome *c* from *Bacillus pasteuri*. (b) 8.4 kDa structure of soluble form of cytochrome b_5_ from *Silicibacter pomeroyi*. Apo (c) and holo (d) forms of bovine cytochrome b_5_ shown in cartoon form with an electrostatic surface of the protein overlaid. Figures prepared using Pymol (Schrödinger Inc.)

Proteins containing a single heme typically have higher ratios of monomer weight/heme than di‐heme cytochromes. The smallest monoheme protein is the 7.1 kDa cytochrome *c* from *Bacillus pasteuri* (Figure 2a).^18^ The majority of *c*‐type cytochromes remain clustered at the lowest part of the ratio, while *b*‐type cytochromes typically have larger sizes. The smallest monomeric *b‐*type cytochrome is the soluble domain from cytochrome b_5_ isolated from *Hadesarchaea archaeon* YNP_N21 (PDB: ID 6NZX), with a molecular weight (Mw) of 8.6 kDa (Figure 2b). The slightly larger size of cytochrome b_5_ proteins compared with cytochrome *c* proteins could be attributed to two properties: First, the thiolate ligands that attach heme to the polypeptide chain enhance the stability of the protein; second, monoheme *c*‐type cytochromes exist in an unfolded state until heme is incorporated.^6^, ^19^ As heme is incorporated into a heme binding site it is necessary for *b*‐type cytochromes to form a stable apo‐form prior to heme incorporation. The structure of the apo form of cytochrome b_5_ has been determined and shows an open hydrophobic pocket ready for heme insertion. Once heme binds to the apoprotein the pocket closes, and the surface of the structure becomes negatively charged.^20^

The globin‐like proteins have an average Mw grouped around 16 ± 3 kDa, while the thiolate ligated family of cytochromes typically have an average Mw of 46 ± 9 kDa. Thus, the average Mw of electron transfer cytochromes are less than for globins or other cytochromes. This could be linked to function, as globins and catalytic cytochromes require additional peptide to create a substrate binding pocket.

The smallest *b*‐type cytochrome with a cysteine ligand was the dimer heme peroxygenase from the fungus *Marasmius rotula* (PDB ID: 5FUK). These peroxygenases are secreted by fungi to degrade lignocellulose using peroxide secreted from fungi. The active site contains several characteristics of conventional P450s, but do not require interaction sites for electron transfer partners, which may explain their smaller size.

### 1.2 Di‐heme *cytochromes*

**Figure 3:**
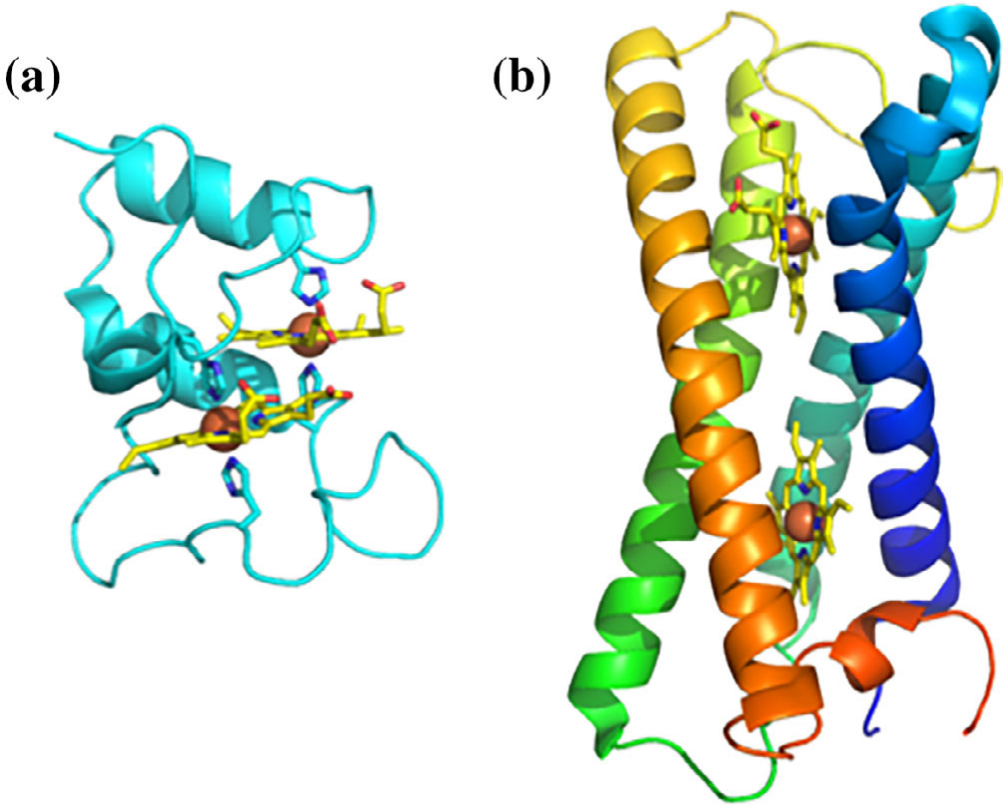
Examples of di‐heme cytochromes. (a) Di‐heme cytochrome c from *Geobacter sulfurreducens*. (b) Cytochrome b561 superoxide oxidase from *Esherichia coli*

There are substantially fewer proteins that contain two hemes, and of these only 40% are *b*‐type, with the remaining 60% belonging to the *c*‐type cytochrome family. There are no proteins with two hemes having cysteine ligation, and only one globin, which is a sensor from *Bordetella pertussis* that is proposed to allow allosteric responses to oxygen binding.^21^ The smallest diheme protein is a *c*‐type cytochrome, called DHC2 from *Geobacter sulfurreducens*, which has a monomer weight/heme ratio of 4.6 kDa/heme^22^ (Figure 3a). In contrast the smallest di‐heme *b*‐type cytochrome is a transmembrane superoxide oxidase from *Esherichia coli* with a much higher ratio of 11.1 kDa/heme^23^ (Figure 3b). This protein is part of the cytochrome b561 family of di‐heme *b‐*type cytochromes. These are transmembrane cytochromes that contain two *bis‐*histidine coordinated hemes packed within the hydrophobic transmembrane helical bundles. These are located near opposing sides of the membrane interface and are often part of electrogenic ET systems.^24^

The transmembrane location consequently makes the di‐heme *b*‐type cytochrome larger than the small monoheme *b*‐type cytochromes that are responsible for single electron transfers to electron donors. The remaining di‐heme *b*‐type proteins are heme binding proteins involved in cytochrome *c* maturation, where the hemes lie stacked parallel inside the protein.

### 1.3 *Tri‐heme cytochromes*

There are few representative structures of cytochromes containing three hemes in the database, namely the 4 *c‐*type and 1 *b‐*type cytochromes. The smallest tri‐heme protein is cytochrome *c*_7_ from *Desulfuromonas acetoxidans* with a ratio of 2.4 kDa/heme. This peptide/heme ratio appears to be at the minimum limit as structures with increasing numbers of hemes are either at or above this ratio. Consequently, the trimeric cytochrome *c*_7_ heme configuration represents a structurally minimized form of heme packing. Cytochrome *c*_7_ variants are found in other bacteria, including the Ppc group of cytochromes from *G. sulfurreducens*, which contains five homologous *c*_7_ proteins PpcA, PpcB, PpcC, PpcD, and PpcE, that are reported to be involved in electron transfer between the cytoplasmic and outer membrane.

The only *b‐*type cytochrome structure with three hemes currently known is the flavohemoglobin from *Alcaligenes eutrophus*, a globular cytoplasmic protein containing three *b*‐ hemes and an FAD molecule.^25^ The exact role of this cytochrome has been elusive, but has been linked to detoxification of reactive nitrosative and oxidative species in the cytoplasm.^26^

### 1.4 *Multiheme (4+) cytochromes*

The only structurally characterized proteins with more than three hemes are prokaryotic *c‐*type cytochromes. This indicates that proteins containing multiple hemes are incorporated as covalently attached hemes, and that cytochromes with four or more hemes are all located outside of the bacterial cytoplasm. This appears logical as hemes can be packed within a polypeptide chain more efficiently if covalently bound when compared to noncovalently bound hemes. This allows for effective use of polypeptide to build a cytochrome containing multiple hemes with active sites and interfaces for electron exchange. This compartmentalization also means that, for bacteria that use networks of electron transfer proteins, electrons are kept out of the cytoplasm in order to minimize production of radical species and prevent damage to DNA and regulatory proteins.

**Figure 4:**
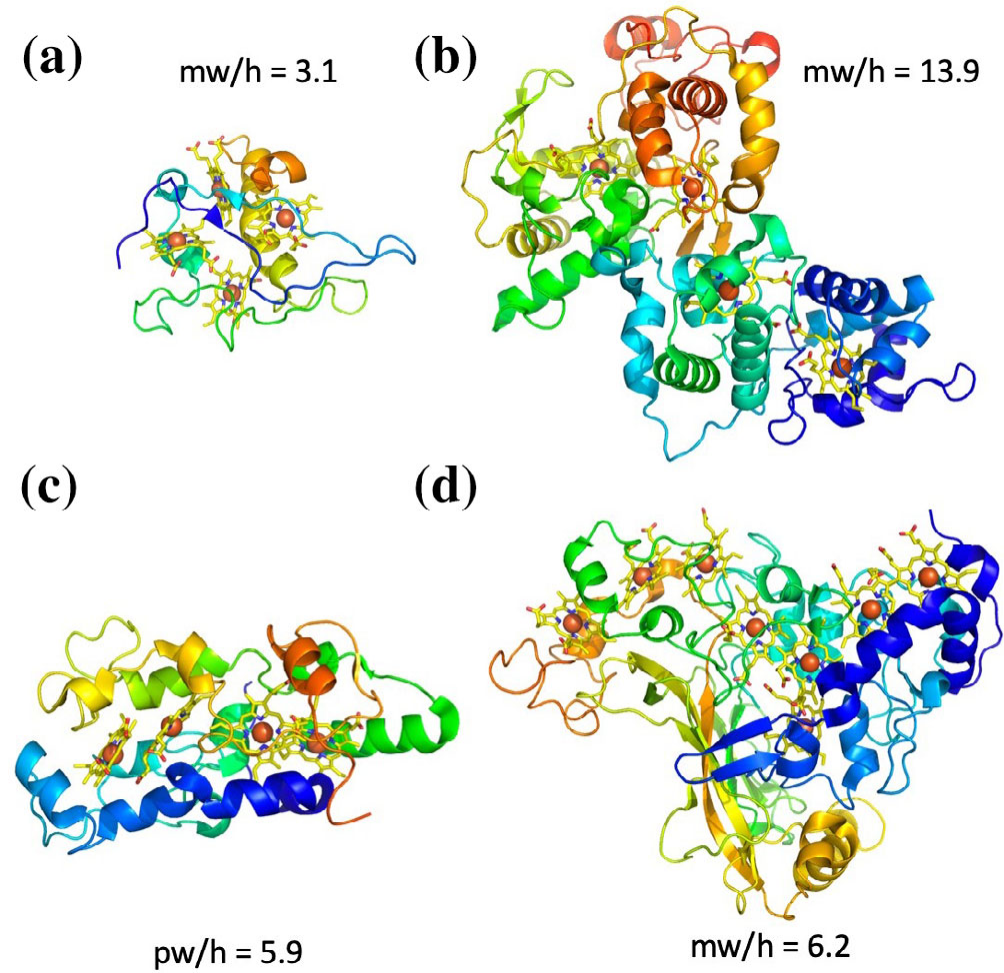
Examples of multiheme cytochromes with different protein weight/heme ratios (mw/h). (a) peptide minimized tetraheme cytochrome *c*_3_ from *Desulfomicrobium baculatum* 29). (b) Peptide replete Thiosulfate dehydrogenase (TsdBA) from *Marichromatium purpuratum*.^30^ (c) *Nitrosomas* electron transfer protein.^27^ (d) Octaheme tetrathionate reductase.^28^ All cytochromes are depicted in cartoon format with yellow hemes contain orange iron atoms

There are currently no structures available for cytochromes that contain 7, 13, 14, 15, or more than 16 hemes per monomer, despite examples of amino acid sequences containing up to 85 CXXCH motifs being predicted in the genome of cytochrome replete iron‐oxidizing bacteria.^14^ The structures of these multiheme *c‐*type cytochromes can be divided into two different groups depending on their relative protein weight /heme ratio (Figure 1). “Peptide minimized” cytochromes have a ratio of less than 5 kDa/heme and are all electron transfer proteins with little secondary structure, while “peptide replete” cytochromes have a ratio greater than 7 kDa/heme and are all catalytic proteins with substantial secondary structure. Between these ratios there is some ambiguity, for instance the tetraheme electron transfer protein *c*_554_ from *Nitrosomas europea* has a ratio of 5.9 kDa/heme^27^ while the octaheme tetrathionate reductase from *Shewanella oneidensis* has a ratio of 6.1 kDa heme.^28^ The higher weight/heme ratio for *c*_554_ is due to having significant secondary structure, presumably to allow for tightly controlled electron transfer between ammonium monooxygenase and the hydroxylamine oxidoreductase (HAO),^27^ while the tetrathionate reductase has less secondary structure than other members of the HAO cytochrome class (Figure 4).

**Figure 5:**
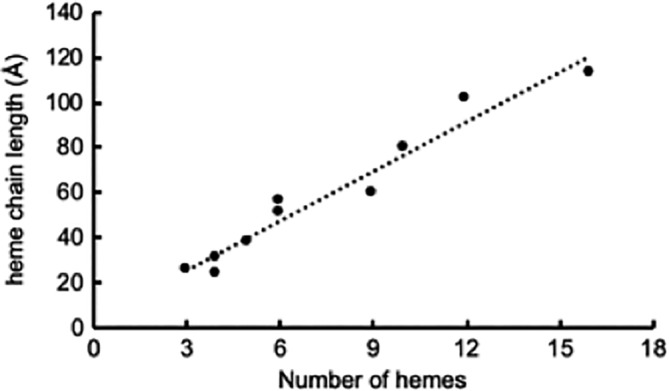
Correlation between heme number and maximum distance between terminal hemes of “peptide minimized” cytochromes containing multiple hemes. Peptide minimised cytochromes were identified in Figure 1 as having a peptide weight: heme ratio of <5 kDa/heme. The line is a linear fit with a r^2^ of .94

The biologically relevant conformations of peptide minimized cytochromes are monomeric and proposed to be involved in electron transfer, electron scavenging, or electron storage. In contrast, many of the peptide replete cytochromes are catalytic and consist of dimeric or higher‐order complexes, such as the 24 *c*‐type heme HAO complex made of three octaheme cytochromes,^31^ or the 192‐heme hydrazine dehydratase complex made from 24 subunits each containing eight hemes.^32^ The peptide minimized cytochromes have hemes aligned in pairs that are closely parallel or perpendicular; this allows for efficient electron transfer across the heme chain within the cytochrome. For these cytochromes there is a linear correlation between the number of hemes and the edge to edge length of the cytochrome (Figure 5). This suggests that in order to pass electrons across the ~ 200 Å width of the periplasm in Gram negative bacteria, a cytochrome would need to contain approximately 27 hemes. Gene clusters containing periplasmic cytochromes with 21–24 hemes have been identified in iron oxidizing bacteria and these might be capable of bridging the inner and outer membranes.^14^ These gene clusters include the 86 heme cytochrome from *A. oligotrophica* S58 in an operon with a porin. This cytochrome has a predicted peptide Mw of 255 kDa, and would have a peptide weight/heme ratio of 2.86, grouping it in the peptide minimized cytochrome family. Based on the linear correlation between heme chain length and heme number from Figure 5, this suggests that the cytochrome would have a heme chain length of 630 Å. It seems unlikely it would simply be involved in electron transfer across the periplasm, it is possible that it connects to the periplasm and then passes out through a porin into the extracellular environment.^14^

## 2 MULTIHEME CYTOCHROMES INVOLVED IN EXTRACELLULAR ELECTRON TRANSFER

While eukaryotes and many bacteria contain genes for only a few *c*‐type cytochromes and typically only express these under very specific conditions, some bacteria are capable of expressing a large number of different cytochromes with high numbers of hemes. In particular, bacteria of the genera of *Shewanellacea* and *Geobacteracea* have been the subject of study for the past three decades due to their ability to reduce a broad range of extracellular substrates, including insoluble Fe(III) and Mn(IV) minerals. They are linked to the broad range of *c*‐type cytochromes that are expressed within the periplasmic compartment and on the surface of the cell. The interactions between the different cytochromes have been studied for over a decade and substantial progress has been made in elucidating the electron transfer networks involved in coupling cytoplasmic respiration to the reduction of extracellular substrates. These systems have evolved different mechanisms to overcome the challenges of extracellular electron transfer: namely 1) electron transfer from the quinol pool in the cytoplasmic membrane to electron carriers in the periplasm 2) electron transfer across the outer membrane or cell wall and then 3) electron transfer from cytochromes on the cell surface into extracellular substrates. For the remainder of this review we shall look at how progress has been made into understanding how different organisms undertake extracellular electron transfer.

## 3 THE OUTER MEMBRANE CYTOCHROMES OF *SHEWANELLA ONEIDENSIS*

**Figure 6:**
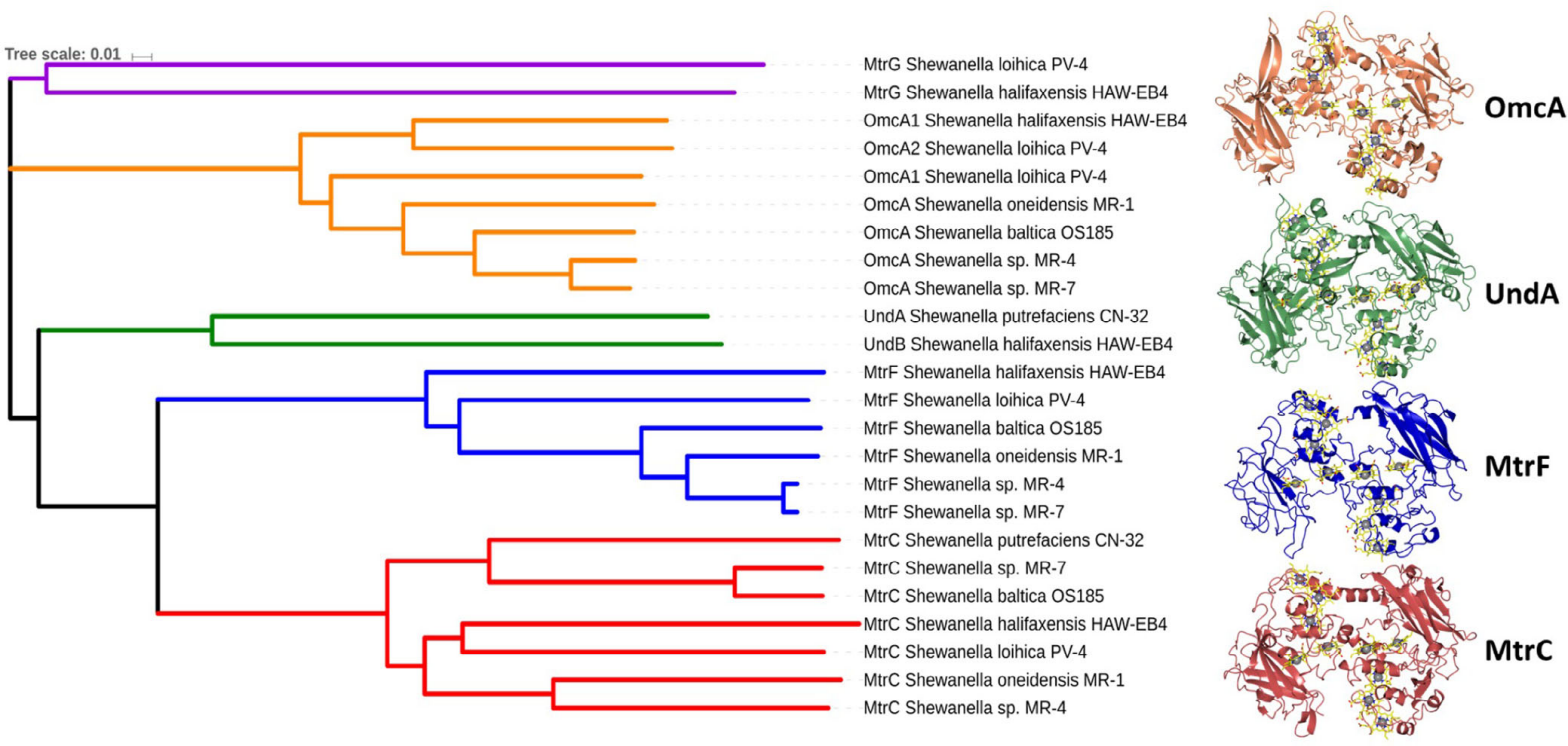
Multiheme cytochromes involved in extracellular electron transfer in *Shewanella oneidensis*. The tetraheme cytochrome CymA (yellow) oxidizes quinol to quinone and transfers electrons via periplasmic cytochromes, such as small tetraheme cytochrome (STC, purple) and fumarate reductase (FccA, pink), to the decaheme cytochrome MtrA (blue) that forms a tight complex with the predicted porin MtrB (green). MtrA passes electrons to the cell surface localized decaheme cytochrome MtrC (red) that forms a tight complex with MtrAB. MtrC serves as a terminal reductase and passes electrons to extracellular acceptors such Fe(III) minerals that is, hematite

In the absence of oxygen, the Gram‐negative bacteria *Shewanella* is able to transfer electrons from the quinol pool in the inner membrane to solid‐phase electron acceptors located outside of the cell. In order to achieve this, a tetraheme quinol dehydrogenase, CymA, oxidizes reduced quinols and transfers these electrons to periplasmic cytochromes, such as the small tetraheme cytochrome (STC) and fumarate reductase (FccA). These soluble cytochromes can act as electron shuttles, moving electrons across the periplasm to the MtrCAB complex embedded in the outer membrane (Figure 6).^33^

The MtrCAB complex consists of a decaheme c‐type cytochrome, MtrA, a predicted 28 β‐strand porin, MtrB, and a cell surface localized decaheme c‐type cytochrome MtrC. MtrA is proposed to be embed within the MtrB porin and form a 10‐heme wire connecting the periplasmic and extracellular environments (Figure 6).^34^ Gene knockout experiments have demonstrated that MtrAB porin‐cytochrome module is capable of directly transferring electrons to soluble electron acceptors at the cell surface (i.e., iron (III) citrate) but is unable to reduce solid phase minerals (i.e., hematite).^35^ The ability to reduce soluble extracellular electron acceptors indicates that the MtrAB “module” is the minimal complex required for electron transfer across the bacterial outer membrane. The cell surface localized decaheme cytochrome MtrC is required to transfer electrons from MtrAB to solid‐phase minerals such as insoluble iron oxides.^35^

MtrC forms a tight complex with MtrAB and the molecular envelope of an intact MtrCAB complex has been resolved by small angle neutron scattering (SANS).^36^ The derived molecular envelope of MtrCAB has dimensions of approximately ∼170 × 60 × 45 Å and reveals MtrC to extend approximately 70 Å above the membrane surface. The dimensions of the molecular envelope support the model that MtrA spans MtrB and extends approximately 30 Å into the periplasm. These experiments suggest that MtrA is unable to span the distance across the periplasm between the inner membrane and outer membrane (ca. 200 Å) but instead receives electrons from CymA via periplasmic cytochromes (e.g., STC and FccA) which act as electron shuttles.

Porin‐cytochrome complexes with similar modular configurations to MtrCAB have been identified in several bacterial species including *G. sulfurreducens* and *Anaeromyxobacter dehalogenans*.^37^, ^38^ Porin‐cytochrome complexes lacking a homologue of the cell surface localized component, MtrC, have also been identified in iron‐oxidizing bacteria including *Sideroxydans lithotrophicus* and *Rhodopseudomonas palustris*.^39^, ^40^

**Figure 7:**
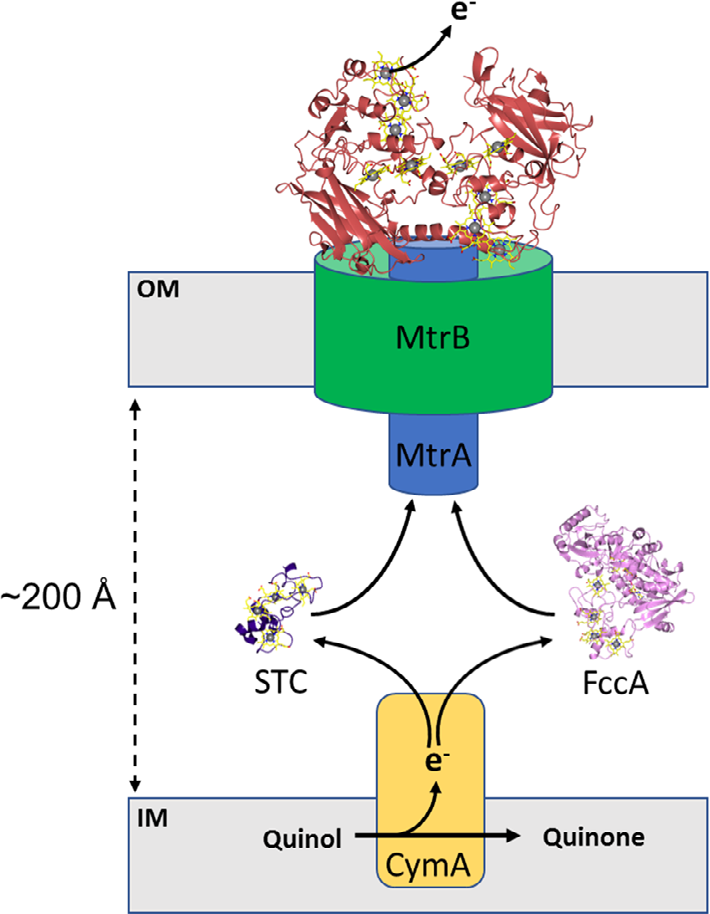
Phylogenetic alignment of the outer membrane cytochromes encoded within the mtr gene clusters of *Shewanella oneidensis* MR‐1, *Shewanella* sp. MR‐4, *Shewanella loihica* PV‐4, *Shewanella putrefaciens* CN‐32, *Shewanella halifaxensis* HAW‐EB4, *Shewanella* sp. MR‐7, and *Shewanella baltica* OS185. Amino acid sequences were aligned and the phylogenetic tree generated with Clustal Omega.^43^ The figure was generated utilizing the interactive tree of life server^44^

In addition to MtrC, the mineral respiring species within the *Shewanellacea* genus express a range of additional cell surface localized cytochromes that are capable of supporting extracellular electron transfer.^35^, ^41^, ^42^ So far, these outer membrane cytochromes (OMCs) can be differentiated into four separated “clades” that consist of the aforementioned decaheme cytochrome MtrC, the decaheme cytochromes MtrF and OmcA, as well as the 11‐heme cytochrome UndA (Figure 7). The cell surface cytochromes of *Shewanella* are localized to the cell surface though secretion by the type II secretion system and anchor to the cell surface through an acylated N‐terminal cysteine.^45^

MtrF forms part of an operon encoding MtrA and MtrB homologues (MtrD and MtrE respectively). Subsequently, MtrF is proposed to form a MtrDEF porin‐cytochrome complex. The requirement for *Shewanella* to possess multiple porin‐cytochrome complexes is unclear; however, expression of MtrDEF has been shown to be upregulated in *Shewanella* biofilms.^46^ In contrast to MtrC and MtrF, the decaheme cytochrome OmcA does not belong to an operon encoding MtrAB homologues and as such is not thought to form a complex with a dedicated porin‐cytochrome. The undecaheme cytochrome UndA, substitutes for OmcA in a number of *Shewanella* genomes.^47^

The structures of the outer membrane cytochromes of *Shewanella* have been resolved.^48^, ^49^, ^50^, ^51^ The structures reveal that although the four major clades of outer membrane cytochromes share less than 35% sequence identity, they display a high degree of structural conservation both in domain organization and arrangement of *c*‐type hemes within the structure. Each cytochrome is comprised of four domains that consist of an N‐terminal split β‐barrel (domain I), a penta‐heme domain (domain II), a second split β‐barrel (domain III) and then either a penta‐ or hexaheme (UndA) C‐terminal domain (domain IV). The four domains are arranged in such a way that the two multiheme domains are in the middle of the structure with the two β‐barrel domains flanking each side and the hemes within the two multiheme domains form a continuous network with the iron atoms of adjacent hemes all being within 10 Å of each other.

Despite low sequence homology, the overall conformation of the 10 core hemes is well conserved across the four different OMC clades, with only the terminal hemes at opposite ends of the staggered cross showing variability in their positions. The role of the β‐barrel domains is unclear, but it may be to do with conferring rigidity on the structure, preventing the tightly packed multiheme chain from collapsing on the surface of the cell.

*Shewanella* secretes small redox active molecules, flavins, into the extracellular environment where they are proposed to either serve as redox shuttles between cytochromes on the cell surface and electron acceptors located too distant for direct electron transfer, or where they potentially serve as redox cofactors of the outer membrane cytochromes.^47^, ^52^, ^53^

## 4 THE OUTER MEMBRANE CYTOCHROMES OF GEOBACTER

Despite intensive research, the mechanism that is used by *Geobacter* to transfer electrons into solid surfaces is still poorly understood, in part because there are a broad range of cytochromes expressed that have overlapping functions. Most recently these have been proposed to allow *Geobacter* to respond more efficiently to a range of different redox potentials.^54^

While the *Shewanellacea* typically use a single quinol dehydrogenase, CymA, during anaerobic respiration there are at least two quinol dehydrogenases in *Geobacter* involved in anaerobic respiration. Both are constitutively expressed and one, ImcH, is required for respiration of extracellular substrates with a potential > −100 mV versus Standard Hydrogen electrode (SHE). The other, CbcL, is involved in respiration of substrates at lower potentials.^55^, ^56^

The two quinol dehydrogenases then transfer electrons into the periplasmic cytochrome pool. In *Geobacter* there are no obvious flavocytochomes like the fumarate reductase FccA of *S. oneidensis*, or tetraheme electron shuttles like STC. Instead there are five tri‐heme *c*_7_ type cytochromes that have high homology to one another, these are PpcA, PpcB, PpcC, PpcD, and PpcE. The members of the Ppc cytochrome family have highly conserved structures and cover a similar potential range, but have subtle differences in the redox potentials of their individual heme groups.^56^ This may alter the flux of electrons through the periplasm in response to different extracellular electron acceptors.^57^, ^58^

Like *Shewanella* a porin‐cytochrome complex has been identified that is predicted to transfer electrons across the outer membrane, but there are at least five other gene clusters encoding distinct porin‐cytochrome complexes in *Geobacter sulfurreducens PCA*.^59^ Two of which have been shown to be functionally active and one that has been isolated and shown to transfer electrons across a lipid bilayer in vitro.^38^, ^60^ Unlike the *S. oneidensis* porin cytochrome complex, these are comprised of an 8‐heme periplasmic cytochrome, a transmembrane porin and a 12‐heme cytochrome on the cell surface. These cytochromes share no homology with any structurally resolved cytochromes, suggesting that the arrangement of hemes passing through the outer membrane will be different.

In contrast to *Shewanella*, the *Geobacter* species do not appear to use soluble electron shuttles but rely on direct contact between cells or long distance contact generated by nanowires to deliver electrons.^61^ A number of proteins have been shown to be essential for electron transfer from the surface of *Geobacter* to extracellular acceptors that include Fe(III) minerals and carbon electrodes. The first outer membrane cytochrome to be structurally characterized from *Geobacter* was OmcF, an analogue of cytochrome c_*6*_ from photosynthetic algae.^62^ Despite the structural similarity, OmcF is substantially different to cytochrome c_6_. Normally cytochrome c_*6*_ is involved in photosynthesis, where it functions as an electron shuttle between cytochrome b_6_f and photosystem 1 in cyanobacteria. However, in *Geobacter* the extracellular OmcF is tethered to the outer membrane by a lipid anchor and has redox midpoint potential of +100 mV versus SHE.^63^ This potential is lower in OmcF than in cytochrome c_6_, but higher than the midpoint potential of most other characterized *Geobacter* cytochromes, making it unlikely to be an electron shuttle involved in extracellular electron transfer. The most compelling evidence for a role for OmcF is that its expression is linked to the expression of two other outer membrane cytochromes, making it either a regulator of extracellular expression, or is involved in cytochrome transport across the outer membrane.^64^

**Figure 8:**
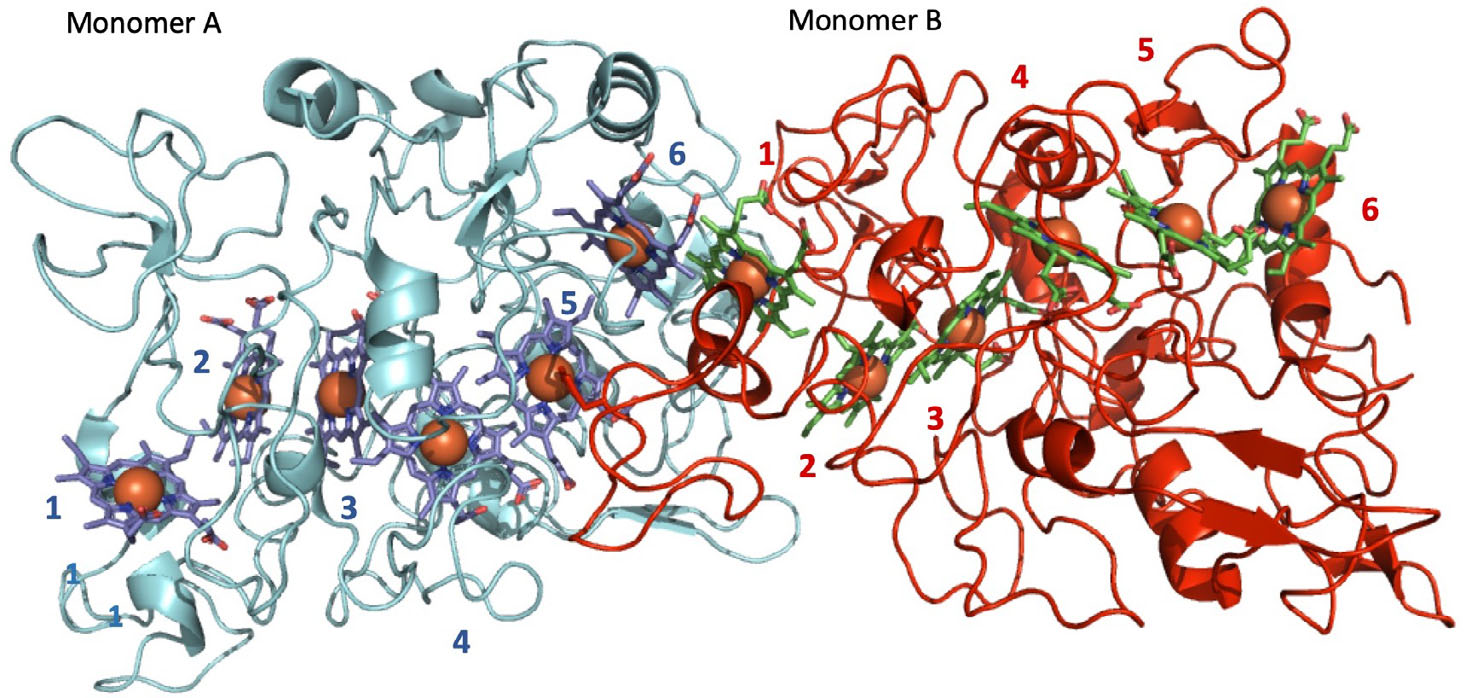
Cryo‐EM structure of OmcS adapted from PDB_ID 6EF8^65^ to show the two terminal ends of OmcS and the interface between OmcS monomers. Two OmcS monomers are shown in cyan (a) and red (b), with heme groups for respective monomers colored blue and green. The hemes of each monomer are numbered according to the position of the CxxCH motif on the amino acid sequence. Heme 1 of monomer A and hemes 5 and 6 of monomer B are exposed. The monomer B histidine ligand that coordinates monomer A heme 5 is shown

The outer membrane hexaheme cytochrome OmcS has long been recognized as being important for reduction of extracellular substrates, and its structure has recently been determined through Cryo‐EM on isolated filaments taken from the surface of *G. sulfurreducens*.^65^, ^66^ These reveal that OmcS is a polymeric cytochrome where the OmcS monomers form into a continuous linear chain. All six hemes within the chain are *bis*‐His coordinated, preventing ligand association and allowing for rapid electron transfer between adjacent hemes by limiting conformational rearrangement on reduction. The histidine for heme 5 is provided from the peptide chain of an adjacent OmcS monomer, making heme ligand assembly part of the polymerization process (Figure 8).

The heme chain in OmcS consists of alternating parallel (offset facing) and perpendicular (T‐junction) heme pairs. This arrangement of hemes is often seen in multiheme cytochromes where electron transfer is necessary. The distances between the porphyrin rings, approximately 3.5 Å for parallel hemes and 6 Å for T‐junction heme pairs allow for rapid transfer of electrons between adjacent hemes. It is unclear how, if at all, the two ends of the OmcS pili are capped to allow for electron exchange between the periplasm and extracellular substrate, but the OmcS nanowire appears optimized for electron exchange between adjacent hexaheme monomers without a clear electron ingress/egress site for cytochromes within the polymeric chain.^65^

The relative molecular weight:heme ratio is 7.5 kDa/heme, which is higher than that of simple electron transporters such as STC and NrfB, and similar to catalytic enzymes such as HAO (7.5) As the OmcS hemes are buried within the structure this suggests that the OmcS polymers are not optimized for nonspecific electron transfer through the sides of the OmcS polymer but are insulated, perhaps to allow for effective conduction away from the cell surface, rather than reduction of nearby extracellular substrates. The unusual coordination of heme 5 means that the terminal monomer of the OmcS heme chain will have a pentacoordinate heme, giving directionality to the OmcS polymer (Figure 8). This terminal monomer could therefore contain a catalytic site for Fe(III) (hydr)oxide reduction, or attach to a membrane anchor on the *Geobacter* surface that connects the polymeric wire to the extracellular interface.^65^

## 5 THE CELL‐SURFACE CYTOCHROMES OF GRAM‐POSITIVE BACTERIA

**Figure 9:**
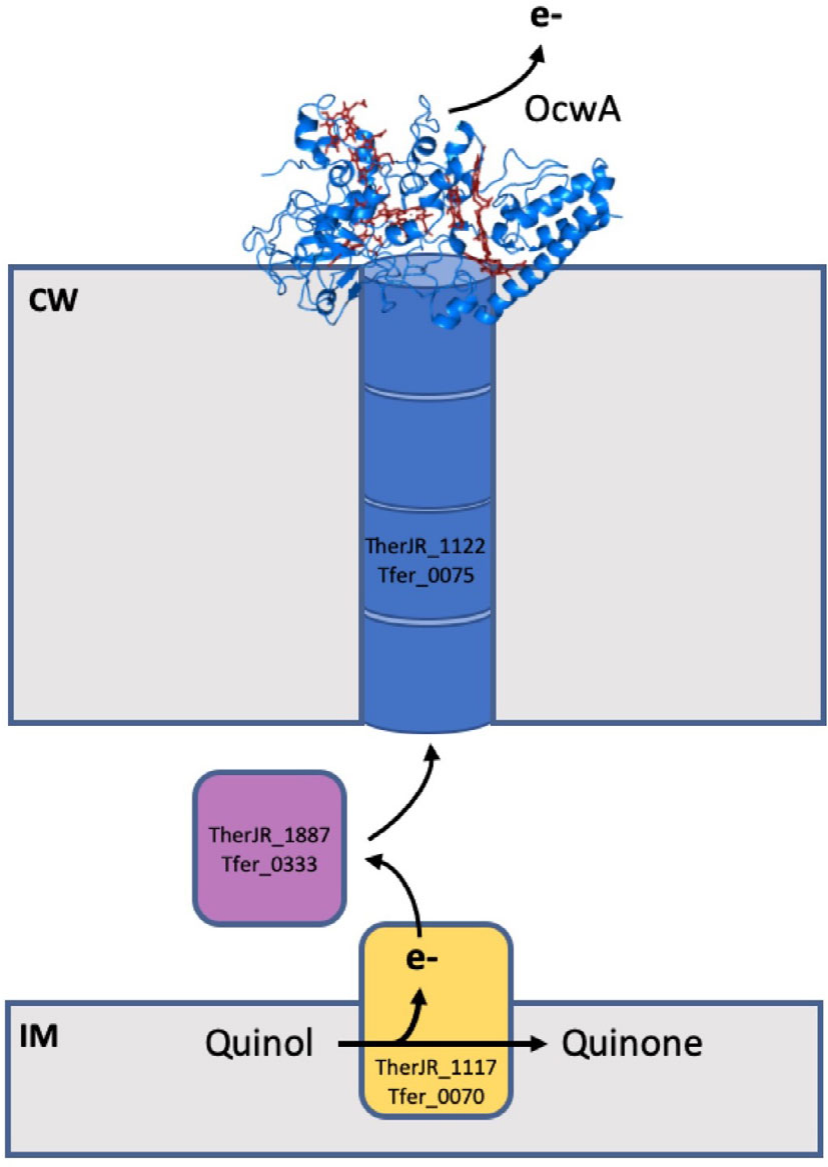
Multiheme cytochromes involved in extracellular electron transfer in *Thermincola*. The inner membrane decaheme cytochrome TherJR_1117 (Tfer_0070) oxidizes quinol to quinone and transfer electrons to the decaheme cytochrome TherJR_1887 (Tfer_0333), that then transfer electrons to the peptidoglycan embedded hexaheme cytochrome TherJr_1122 (Tfer_0075). This cytochrome transfers electrons across the cell wall to the terminal reductase OcwA (TherJR_2595 and Tfer_3197), which then passes the electrons to extracellular electron acceptors, such as electrodes or iron minerals

In contrast to Gram‐negative organisms, such as *Shewanella* and *Geobacter*, Gram‐positive bacteria are characterized by possessing a cell wall composed of peptidoglycan, sugars, phospholipids, peptides, amino acids, and glycoprotein.^67^ It was proposed that the thick peptidoglycan layer would prevent Gram‐positive bacteria from performing extracellular electron transfer.^67^, ^68^, ^69^, ^70^ However, the isolation of *Thermincola potens* strain JR in a current‐producing MFC operating at high temperature altered this view.^71^ Later, *T. ferriacetica*, that shares 99% similarity with *T. potens*, was also shown to be capable of transferring electrons from acetate to the anode of an MFC to generate electric current.^68^ Like most electroactive organisms, *T. potens* has several genes coding for multiheme cytochromes,^13^, ^72^ with several of them proposed to participate in extracellular electron transfer processes^73^ (Figure 9).

The putative extracellular electron transfer pathway was proposed to be composed of four proteins: a decaheme cytochrome (TherJR_1117 in *T. potens* and Tfer_0070 in *T. ferriacetica*) that is anchored to the inner membrane and is proposed to receive electrons from the menaquinone pool; a periplasmic decaheme cytochrome (TherJR_0333 in *T. potens* and Tfer_1887 in *T. ferriacetica*) proposed to transfer electrons within the periplasmic space of *Thermincola*; a hexaheme cytochrome, proposed to be embedded in the peptidoglycan (TherJR_1122 in T. potens and Tfer_0075 in *T. ferriacetica*), and a nonaheme cytochrome proposed to be the terminal reductase localized at the cell surface of the bacteria (TherJR_2595 in *T. potens* and Tfer_3197 in *T. ferriacetica*) (Figure 9). From these, only the cell‐surface multiheme cytochrome, named outer cell‐wall cytochrome A (OcwA) was characterized in detail.^74^

OcwA, in contrast to other known terminal reductases of insoluble compounds, contains hemes with different coordination environments. It contains five hemes with the typical bis‐histidine axial coordination (hemes 1, 3, 4, 6, 7, and 8), one heme with histidine‐methionine coordination (heme 9), and two hemes with a histidine as the proximal ligand and an open coordination side as the distal position (hemes 2 and 5). It was shown that these two high‐spin hemes, located at opposite ends of the nine heme arrangement may work as the putative active sites for substrate binding, which is a novelty within the family of multiheme cytochromes.^74^

Although the organization of the hemes in OcwA vaguely resemble the “staggered cross” of the four major clades of outer membrane cytochromes from *Shewanella*, it clearly follows a different design. The heme arrangement is similar to that of NrfA family of proteins. While heme 1 to 4 align with the hemes of the tetraheme cytochrome *c*_554_ from *Nitrosomonas europaea* with heme 2 as the active site, hemes 5 to 9 can be superimposed to the NrfA heme core structure with heme 5 of OcwA as the active site. Furthermore, hemes 1 to 4 and 6 to 9 align to the heme core structure of the sulfite reductase MccA, with heme 2 acting as the active site. OcwA was shown to reduce nitrite and hydroxylamine, as well as iron oxides.^74^ This suggests a multifunctional role of this protein in the respiratory process of *Thermincola* spp., allowing these Gram‐positive bacteria to grow and survive in environments with various terminal electron acceptors.

## 6 FUTURE PERSPECTIVES

Over the last two decades, the substantial increase in structural and biochemical data on multiheme cytochromes has allowed a much greater understanding of their role in facilitating electron transfer within the cell. Substantial in vitro and in vivo research on the model organisms detailed earlier reveal that multiheme cytochromes with low monomer weight: Heme ratios do not form stable electron transfer networks but instead rely on transient interactions for electron transfer, thereby generating dynamic networks of electron transfer proteins that move electrons from the quinol pool to the cell surface. There is a large amount of apparent redundancy in these systems, with multiple quinol dehydrogenases and highly homologous periplasmic cytochromes in the *Geobacter* extracellular electron transport system, and a range of different extracellular cytochromes in the *Shewanella* extracellular electron transport systems. We prefer to think in terms of “overlapping function”, rather than “redundancy,” as the energetic costs of maintaining these systems in a genome must provide selective advantage to an organism. The challenge is for researchers to strive to understand the overlapping roles of these proteins. While these are not yet clear they may, for example, allow the bacteria to access a greater range of substrates by shifting the redox potentials of the electron transfer network in response to the potential at both the cytoplasmic membrane and extracellular surface. Piecing together how electrons are distributed across intermediate distances within the periplasm or intermediate space will help to shed further light on these complex, multicomponent systems.

## FUNDING

This research was supported by the Biotechnology and Biological Sciences Research Council grants BB/P01819X/1, BB/K009885/1, BB/L023733/1 and by Fundação para a Ciência e a Tecnologia (FCT) Portugal (PTDC/BBB‐BQB/4178/2014, and PTDC/BIA‐BQM/30176/2017), by Project LISBOA‐01‐0145‐FEDER‐007660 (Microbiologia Molecular, Estrutural e Celular) funded by FEDER funds through COMPETE2020—Programa Operacional Competitividade e Internacionalização (POCI) and by the European Union's Horizon 2020 research and innovation program under grant agreement No 810856.
